# Decellularized Persian walnut leaf (*Juglans regia*) as a potential wound dressing scaffold: an experimental study

**DOI:** 10.3389/fbioe.2025.1524956

**Published:** 2025-03-04

**Authors:** Mehdi Kian, Seyedeh Sara Hashemi, Amin Derakhshanfar, Gholam-Hossein Darya, Zahra Shahhossein, Mohmmad Jamal Saharkhiz

**Affiliations:** ^1^ Department of Comparative Biomedical Sciences, School of Advanced Medical Sciences and Technologies, Shiraz University of Medical Sciences, Shiraz, Fars, Iran; ^2^ Student Research Committee, Shiraz University of Medical Sciences, Shiraz, Fars, Iran; ^3^ Burn and Wound Healing Research Center, Shiraz University of Medical Sciences, Shiraz, Iran; ^4^ Department of Horticultural Sciences, School of Agriculture, Shiraz University, Shiraz, Iran; ^5^ Medicinal Plants Processing Research Center, Shiraz University of Medical Sciences, Shiraz, Iran

**Keywords:** *Juglans regia*, decellularized leaf, plant-based biomaterials, mesenchymal stem cells, wound dressing, skin, mice

## Abstract

**Introduction:**

Wound dressings often fall short of providing the multifunctional capabilities required for optimal wound healing, such as promoting cell migration, proliferation, and tissue regeneration. Decellularization of plant tissues has gained attention as a potential source of biomaterials for tissue engineering applications due to their favorable characteristics, including pre-existing vascular networks, interconnected porous structure, efficient water transport and retention, high surface area, and a diverse range of mechanical properties.

**Methods:**

This study investigates the feasibility of using decellularized walnut leaves (DWL) as a novel scaffold for wound dressing in a mice model of excisional wounds. The decellularization and bleaching processes were carried out using various chemical agents. DNA and protein quantification and hematoxylin and eosin staining were performed to reveal the successful removal of cells in DWL. Scanning electron microscopy (SEM) was used to indicate that the normal structure of walnut leaves was preserved after chemical decellularization. Chemical characterization was conducted using Fourier-transform infrared (FTIR) and Raman spectroscopy to show the remaining bioactive molecules and components in the structure of DWL.

**Results:**

Comparing tensile strength and surface roughness parameters, surface wettability, swelling, and porosity properties of native and DWL indicated no statistical differences between them. SEM analysis demonstrated that human mesenchymal stem cells excellently attach and proliferate on the DWL. Additionally, the biocompatibility and potential of DWL scaffolds to accelerate wound closure and enhance histopathological scores, collagen deposition, and epithelial thickness were observed in a mice model of excisional wounds.

**Discussion:**

In conclusion, DWL shows promising potential for application as a skin wound dressing due to its biocompatibility, ability to promote cell attachment and proliferation, and efficacy in accelerating wound healing.

## 1 Introduction

Wound healing is a complex and dynamic process that involves a series of coordinated events such as inflammation, proliferation, and remodeling of the tissue (Nguyen et al., 2023; Tottoli et al., 2020). Despite significant advancements in wound care management, finding an effective and affordable wound dressing material remains a challenge in the medical field (Rani Raju et al., 2022; Tottoli et al., 2020). The most common treatment for wounds is the use of a dressing to cover the wound, reduce the risk of infection, and promote healing (Dai et al., 2020; Nguyen et al., 2023). Traditional dressings, such as cotton bandage and gauze have been widely used, but their limitations such as insufficient hemostatic efficacy and non-moist environment have led to the exploration of more advanced wound dressing materials (Farahani and Shafiee, 2021; Rezvani Ghomi et al., 2023).

Tissue-engineered scaffolds are a modern medical strategy for wound dressing (Farahani and Shafiee, 2021). They mimic the 3-dimensional structure and physiological properties of skin tissue and extracellular matrix (Farahani and Shafiee, 2021; Tarrahi et al., 2022; Tottoli et al., 2020). In recent years, decellularized leaves have been gaining attention in the field of tissue engineering due to their use of plant-based materials as a source of scaffold for tissue engineering has been explored in the last decades (Arslan et al., 2023; Bilirgen et al., 2021; Indurkar et al., 2021; Mutra et al., 2023). Various studies have shown that decellularized leaves have the potential to be used as a natural scaffold for tissue regeneration purposes (Bilirgen et al., 2021; Harris et al., 2022; Harris, Lacombe and Zenhausern, 2021).

Decellularization is a process that involves the removal of cellular components from a tissue or organ, leaving behind the extracellular matrix (ECM) or the structural framework of the tissue (Neishabouri et al., 2022). This process in the plant tissues is achieved by several techniques, including chemical, detergent-free, freeze/enzymatic, and supercritical fluid (Bilirgen et al., 2021; Harris et al., 2022; Zhu et al., 2021). Decellularized leaves have several advantages as a biomaterial for tissue engineering applications (Bilirgen et al., 2021; Gorbenko et al., 2023; Mutra et al., 2023; Thippan et al., 2019; Zhu et al., 2021). First, they are readily available and can be easily obtained from a variety of plant species (Zhu et al., 2021). Second, they are biocompatible, meaning they do not elicit an immune response when implanted in the body (Bilirgen et al., 2021). Third, they have a highly organized ECM structure, which provides a template for tissue regeneration (Bilirgen et al., 2021; Zhu et al., 2021). Fourth, they are relatively inexpensive compared to other biomaterials (Zhu et al., 2021). Fifth, which is very important, decellularized leaves scaffolds naturally have a vascular structure that can be used as a perfusion platform for the bioengineering of larger grafts (Bilirgen et al., 2021; Gorbenko et al., 2023; Mutra et al., 2023; Thippan et al., 2019; Zhu et al., 2021).

Furthermore, decellularized leaves have been shown to promote the growth and differentiation of various mammalian cell types, including mouse myoblast cells (Varhama et al., 2019), human dermal fibroblasts (Dikici et al., 2019; Harris, Lacombe, Liyanage, et al., 2021; Phan et al., 2020), human dermal microvascular endothelial cells (Dikici et al., 2019), human mesenchymal stem cells (Adamski et al., 2018; Gershlak et al., 2017; Salehi et al., 2020), human umbilical vein endothelial cells (Fontana et al., 2017; Gershlak et al., 2017), human breast cancer cells (Wang et al., 2020), and hepatocellular carcinoma cells (Ahmadian et al., 2023).

Persian walnut leaf is extensively used as remedies in conventional medicine as well as in pharmaceutical and cosmetic industries as a source of valuable medicinal and healthcare compounds (Jahanban-Esfahlan et al., 2019; Vieira et al., 2017). It possesses phytochemicals, including phenolic compounds, flavonoids, and tannins, which have been reported to have antioxidant, anti-inflammatory, and antimicrobial properties (Delaviz et al., 2017; Jahanban-Esfahlan et al., 2019; Sharma et al., 2022) that are beneficial for wound healing (Amel et al., 2021; Nasiry et al., 2022; Nozohour et al., 2019).

This study aimed to investigate the potential of decellularized walnut leaves for application as a wound dressing. Considering the potential of skin cells to attach and proliferate on decellularized leaf scaffolds based on previously mentioned studies and traditional medicinal uses of walnut leaf and its wound healing properties, the hypothesis of the present study was that DWL can provide a suitable matrix for cell attachment and proliferation of skin cells, thereby promoting wound healing in a mice model of excisional wound.

## 2 Experimental section

### 2.1 Materials

Sodium dodecyl sulfate (SDS), Triton-X100, sodium chlorite, 3-(4,5-dimethyltiazole-2-yl)- 2,5-diphenyltetrazolium bromide (MTT), dimethyl sulfoxide (DMSO), Phosphate-buffered saline (PBS) and from Sigma–Aldrich (Burlington, VT, United States). DMEM medium Fetal bovine serum (FBS) and Pen-Strep solution were purchased from Shellmax (Zhejiang, China). Ketamine and xylazine were purchased from Alfasan (Woerden, Netherlands) Total DNA quantification Kit was obtained from DENAzist Asia Co., (Mashhad, Iran), and total protein extraction and quantification kits were purchased from Arsam Farazist (Orumiyeh, Iran). Simple eye ointment was obtained from Lubratex, Sina Darou, (Tehran, Iran). Deionized water was purchased from Alian Tajhiz (Shiraz, Iran).

### 2.2 Preparation and decellularization of walnut leaves

**FIGURE 1:**
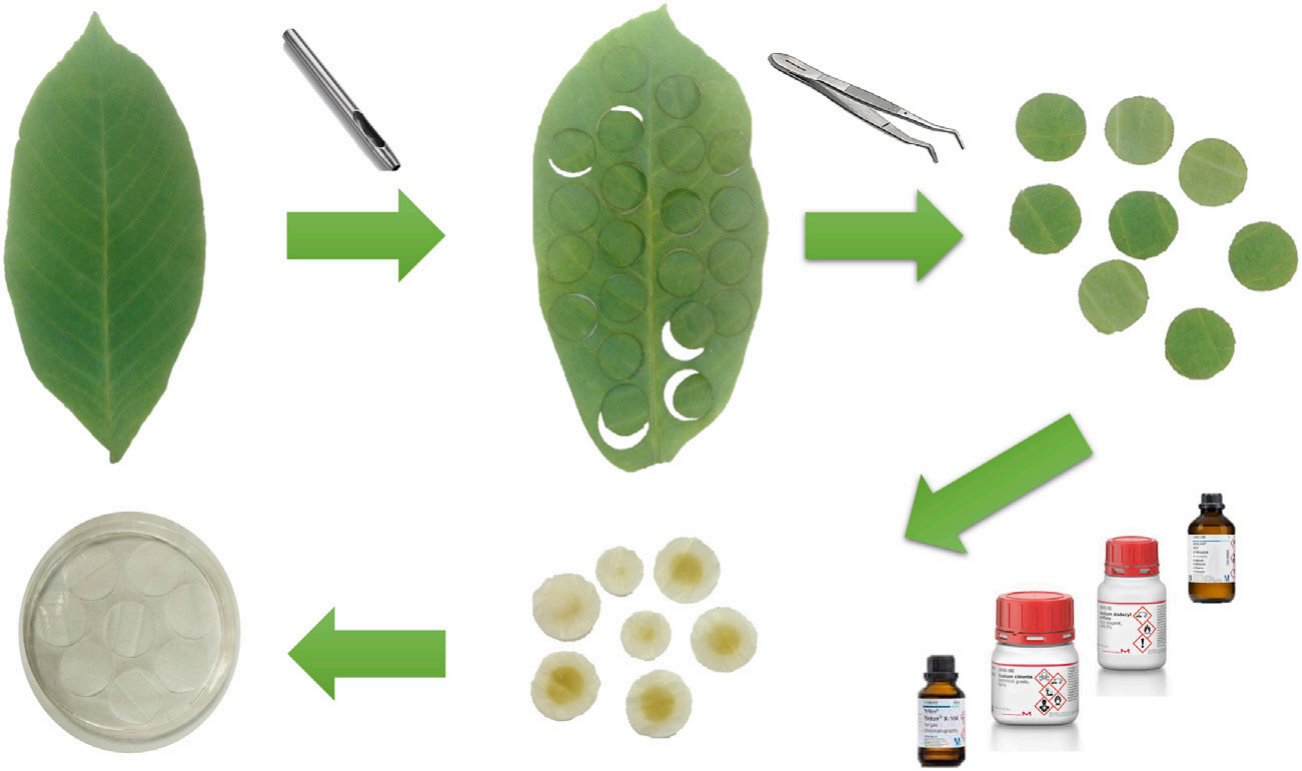
Process of the decellularization of the walnut leaves.

Walnut leaves were collected from Shiraz city (Fars province, Iran). They were identified and authenticated by a botanist from Shiraz University of Medical Sciences (3082-*Jagulans regia L*). First, the walnut leaves were soaked and washed with deionized water. Then, the leaves were punched circularly with different diameters by sterile stainless steel hollow hole punch tools. The decellularization process of circular walnut leaf samples was chemically carried out according to the previously described methods (Adamski et al., 2018; Harris, Lacombe, Liyanage, et al., 2021) with some modifications. In brief, walnut leaves were soaked twice in deionized water (DW) for 5 min and then soaked in n-hexane for 24 h to completely remove the wax coating from the surface of the leaves. Next, the leaves were immersed in 1% SDS for 5 days. During the decellularization process, the walnut leaf samples were incubated at room temperature (20°C–25°C) in a shaking incubator (Fan Azma Gostar, Iran) set to a low speed to prevent damage to the samples. After that, walnut leaves were treated with a solution of 0.1% Triton-X 100% and 10% sodium chlorite in DW water for 48 h (Figure 1). Prepared DWL samples were soaked in PBS twice for 4 h to remove any residual chemical agents. After being air-dried, some samples were kept at - 20°C for further total DNA and protein quantification and the others were kept at room temperature.

### 2.3 Total DNA and protein quantification

Both DWL and Native walnut leaves (NWL) samples were put in a liquid nitrogen bath and ground with a pestle. Fragments were further processed by pulling through a 25-gauge syringe needle 3 times to reduce leaf fragment size. DNA was then measured according to the total DNA quantification kit instruction and the total amount of DNA was assessed using a nanodrop. Similarly, protein was extracted according to the instructions of the kit manufacturer and quantified using the Bradford method. Total protein content in each mg of tissue was assessed using a spectrophotometer (Perkin Elmer, Waltham, MA). The experiments were performed in triplicate.

### 2.4 Histological analysis

DWL and NWL samples were cut into approximately 1 cm squares and processed using an Automatic Tissue Processor (DS 2000/H, Did Sabz, Iran). The prepared paraffin blocks were sectioned into 14 μm thick slices using a microtome (Model DS8402, Iran). The sections were then stained using Safranin O, following the method described by Gershlak et al. (2017). Histopathological slides were examined with a light microscope (Model BX61, Olympus, Japan), and images were captured.

### 2.5 Fourier transform infrared (FTIR) spectroscopy

The functional groups present in the native and decellularized walnut leaves (NWL and DWL) were evaluated by Fourier Transform Infrared Spectroscopy (FTIR, Tensor ІІ FTIR spectroscopy Bruker, Germany). The transmittance was measured in the range of 4,000 to 400 cm^-1^ with a resolution of 4.0 cm^−1^ and eight scans. Five samples from each NWL and DWL were tested.

### 2.6 Raman confocal microscopy

The NWL and DWL samples were prepared for Raman confocal microscopy. The laser power level at 50 mW using the excitation laser wavelength of 532 nm with 100 mV power was used to obtain Raman spectra. We analyzed the Raman spectra at the range of 500–3,100 cm^−1^. The magnification was ×100. Five samples from each NWL and DWL were tested.

### 2.7 Scanning electron microscopy (SEM) and EDX analysis

To investigate the morphology of NWL and DWL samples were coated with gold using a sputter coater (DSR1, Nanostructural Coating Co., Tehran, Iran). After that, SEM and EDX analysis was carried out by a scanning electron microscope (TESCAN Vega3, Brno, Czech Republic) at a voltage of 15 kV. Five samples from each NWL and DWL were tested.

### 2.8 Atomic force microscopy (AFM)

The surface roughness of the NWL and DWL was evaluated by AFM (model ARA AFM, ARA RESEARCH Co., Iran) in contact mode. The surface roughness was obtained by dragging the tip along the surface of the NWL and DWL. Five samples from each NWL and DWL were first fixed on the glass slides with vacuum grease and mounted on a magnetic AFM stage at room temperature. For each sample, force maps were recorded at three different positions. Images were obtained on a 5 × 5 μm surface area and expressed as height and phase images. Also, roughness parameters including the roughness average (Ra), average root mean square average of the profile heights (RMS), average maximum profile peak height, (RPM), and average maximum height of the Profile (Rz) were assessed.

### 2.9 Mechanical properties

The mechanical properties of NWL and DWL were evaluated in wet conditions using a universal testing machine (Santam, Iran) equipped with a 100 N load cell at a displacement rate of 0.1 mm/s and with a preload of 0.1 N. Samples for mechanical testing were cut out in a rectangular shape of 50 mm × 10 mm and immersed in PBS 1X at room temperature for 30 min to rehydrate (Bottino et al., 2010; Nitti et al., 2018). The thickness of wet rectangular specimens was measured using a digital vernier caliper (Model 500–197–20 Mitutoyo, Japan). Five samples from each NWL and DWL were tested and the ultimate tensile strength (UTS) and elongation at break were calculated.

### 2.10 Water contact angle

The contact angles of the NWL and DWL were tested by a contact angle analyzer (Jikan CAG-10 semi-automatic goniometer, Jikan Co., Iran) to evaluate their surface wettability. Five circular 16 mm samples from each NWL and DWL were placed on the glass slides and then, 1 μL of distilled water was dropped on the surface of the leaf samples. After 3 s, images from each droplet on the sample surface were captured and the contact angles were measured using the ImageJ software (version 1.51w, NIH, United States).

### 2.11 Porosity

The porosity of NWL and DWL samples was evaluated by ethanol displacement method based on Archimedes’ principle (Pati et al., 2012). Briefly, five dry samples from NWL and DWL were immersed in anhydrous ethanol for 4 h and weighed as w_d_. The porosity of different scaffolds was calculated according to below formula:

$$\text{Porosity}=\frac{W2-W3-\text{Wds}}{W1-W3}\times 100$$

where W1 is the weight of the falcon tube filled with ethanol; W2 is the weight of the falcon tube including ethanol and the NWL or DWL samples; W3 is the weight of falcon tube when the ethanol saturated samples have been removed from W2; and Wds is weight of the dry samples.

### 2.12 Swelling behavior

The swelling ratio of the NWL and DWL samples was determined at different intervals (30, 60, 120, 180, and 240 min) using Equation 2 based on Ahmadian method (Ahmadian et al., 2023):

$$\text{Swelling ratio }\left(\%\right)=\text{Ws}-\text{Wd}/\text{Wd}\times 100\%$$

where Ws is the weight of the swollen samples and Wd is the dry weight. Five samples from each NWL and DWL were tested.

### 2.13 Cell culture, SEM images, and cytocompatibility test

UV-sterilized circular samples with a diameter of 1 mm were placed in 96-well cell culture plates. The hMSCs (National Cell Bank, Pasteur Institute of Iran, Tehran, Iran) with a cell density of 10^5^ cells/well were seeded on the surface of the samples and cultured in a medium composed of 90 *v/v* % DMEM, 10 *v/v* % FBS, and 1 *v/v* % penicillin/streptomycin. The plates were placed in a cell incubator with 37°C and 5% CO_2_ for culturing for 3, 7, and 14 days. For investigation of cell anchorage and proliferation, samples on day 3 were fixed with glutaraldehyde and evaluated by SEM. The cytotoxicity of the samples was measured by MTT assay on days 3, 7 and 14. Cells were cultured at each time point, the medium was removed and washed with PBS, MTT powder was added, and cells continued to incubate in dark conditions for 4 h. The supernatant was measured by an ELISA microplate reader (BioTek, United States) at 570 nm to investigate the optic density (OD) of the samples. Five samples from each NWL and DWL were used for each time point.

### 2.14 Animals

Forty 8–10-week-old male Balb/c mice were provided by the Center of Comparative and Experimental Medicine of Shiraz University of Medical Sciences, Shiraz, Iran. The animals were housed individually under the 12-h light/dark cycle, at 23°C ± 2°C and 60% humidity, and had free access to standard laboratory food pellets (Pars Animal Feed Co., Iran) and water. Efforts were made to avoid all unnecessary distress to the animals. Housing, anesthesia, and post-operative care were performed in compliance with the Animal Rights Monitoring Committee of Shiraz University of Medical Sciences (IR.SUMS.AEC.1401.003). The number of animals per group was calculated based on the resource equation method (Charan and Kantharia, 2013).

### 2.15 *In vivo* study design

To evaluate the biocompatibility and wound healing potential of the DWL, an experimental Stented excisional wound was created on the mice model. After 2 weeks of adaptations, the animals were anesthetized intraperitoneally with a mixture of ketamine 80 mg/kg and xylazine 5 mg/kg (Harikrishnan, 2021). Lidocaine at a dose of 4 mg/kg (Gargiulo et al., 2012) and meloxicam at a dose of 5 mg/kg (Flecknell, 2018) were administered subcutaneously in mice following induction of the anesthesia. A simple eye ointment was applied to prevent eye dryness in the animals. After shaving the dorsal skin of the mice, a silicone O-ring was sutured on the back of animals by a 4/0 nylon surgical thread (Supalon, SUPA Medical Devices Co., Iran) to the dorsal skin. An excisional wound was surgically created in the dorsal skin of each mouse using a sterilized scissor, and the entire thickness of the skin was removed.

Next, the animals were randomly divided into two groups. Wounds of the mice in one group were grafted with DWL while wounds of the other group had not wound dressing graft and served as the control group. On 3, 7, 10, and 14 postoperative days, five animals from each group were euthanized by inhalation of CO_2_ gas according to the American Veterinary Medical Association guideline (Underwood and Anthony, 2020).

### 2.16 Wound closure analysis

Digital photographs from each wound were taken at the time of surgery and after the euthanasia of mice on the mentioned days. A ruler was placed near the wounds for further standard measurements of wound area in the ImageJ software (version 1.51w, NIH, United States). The wound closure rate was calculated and compared between the groups at each time point using the following formula (Huang et al., 2023):

$$\text{Wound closure}=\frac{\text{Wound}\;\text{area}\;\text{on}\;\text{day}\;0-\text{Wound}\;\text{area}\;\text{on}\;\text{day}\;X}{\text{Wound}\;\text{area}\;\text{on}\;\text{day}\;0}\times 100$$

### 2.17 Histopathological evaluations

Explanted scar tissue and unwounded skin were harvested on days 3, 7, 10, and 14 after the experimental creation of stented excisional wounds on the animals using a sharp scissor. The tissue samples were fixed in 10% formalin., Hematoxylin and Eosin (H&E) and Masson’s Trichrome staining were performed according to the manufacturer’s recommendations. Histopathological slides were evaluated using a light microscope (Model BX61, Olympus, Japan) and their images were captured. A histopathological score was given to each slide based on a scoring system provided in Table 1 by two blind observers (Hashemi et al., 2023). The Scoring is based on multiple factors, including the amount of polymorph nuclear cells, re-epithelialization, amounts of granulation and fibroplasia, granulation and fibroplasia maturation, and neovascularization (Hashemi et al., 2023; Rafati et al., 2018). Also, collagen deposition and epidermal thickness were measured by the ImageJ software.

**TABLE 1:** Histopathological scoring.

Score				
Parameter score	0	1	2	3
PMNL amount	Abundant	Moderate	Scant	None
Re-epithelialization	None	Scant	Moderate	Abundant
Granulation and Fibroplasia Amounts	None	Scant	Moderate	Abundant
Granulation and Fibroplasia Maturation	Immature	Mild Maturation	Moderate Maturation	Fully Matured
Neovascularization	None	Up to 5 vessels per ^a^HPF	6–10 vessels per HPF	More than 10 vessels per HPF

^a^ High power field.

### 2.18 Statistical analysis

GraphPad Prism version 9.5.0 (United States) was employed for the analysis and illustration of data. The normality of the distribution of data was evaluated using Kolmogorov-Smirnov test. The homogeneity of variance was tested using the Brown-Forsythe test. Unpaired-T test was used for analyzing total DNA and protein contents, surface roughness (except Ra), mechanical parameters, WCA, and porosity data. The two-way ANOVA test was performed to analyze data from swelling behavior, MTT assay, wound closure, histopathological score, collagen deposition, and epidermal thickness measurement. The obtained data were presented as mean ± SD. Ra was analyzed using the Mann-Whitney test and the results were expressed as median ± range. The p-values<0.05 were considered as significant.

## 3 Results and discussion

### 3.1 Total DNA and protein content of NWL and DWL

**FIGURE 2:**
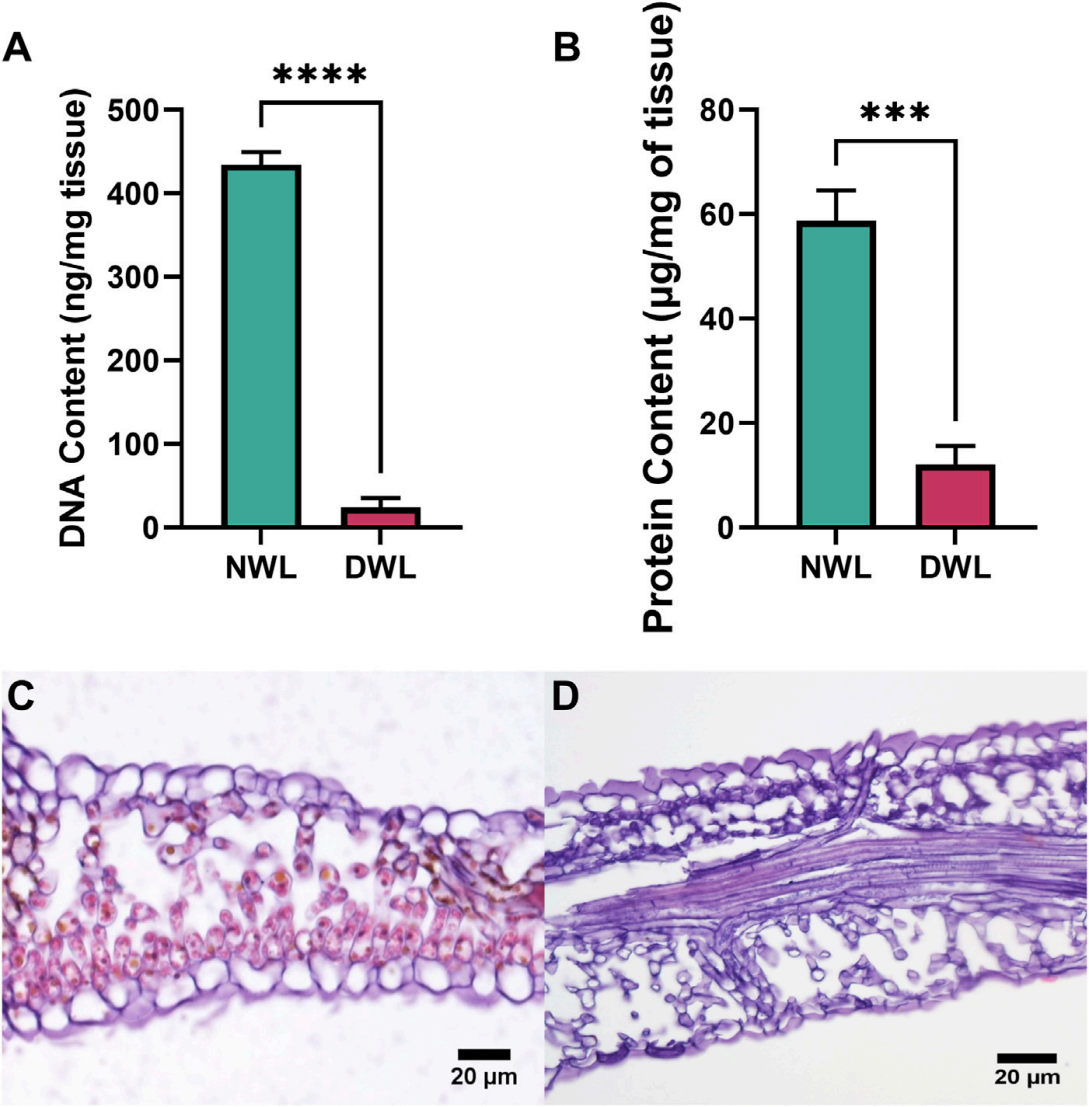
DNA and protein contents in NWL and DWL samples **(A, B)**. Safranin staining image of NWL and DWL scaffolds **(C, D)**.

DNA and protein contents of NWL and DWL are illustrated in Figures 2A, B, respectively. DWL samples had a DNA content of 23.81 ± 11.53 ng and protein content of 10.12 ± 2.09 µg per mg of tissue, respectively, whereas NWL had an amount of 434.02 ± 15.8 ng of DNA and 58.78 ± 5.78 µg of protein per mg of tissue, respectively. Thus, the DNA and protein contents were significantly (P < 0.0001 and P < 0.001, respectively) reduced compared to the NWL, and only 5.48% of the initial DNA and 17.21% of the initial protein from NWL tissue remained in the DWL samples. Histological analysis showed cells with nuclei and chloroplasts in NWL (Figure 2C), neither of which was seen in DWL (Figure 2D).

Effective decellularization is typically confirmed by using staining for nuclear material and quantification of total DNA and protein, as well as characterization of matrix molecule retention (Phan et al., 2020). Both total DNA and protein were significantly reduced in DWL which indicated the decellularization process was conducted well and these findings are in line with the previous studies that had almost similar methods (Gershlak et al., 2017; Harris et al., 2022; Harris, Lacombe, Liyanage, et al., 2021). It was proposed that the amount of less than 50 ng of DNA per mg of dry weight of the ECM of native material is allowed in decellularized biomaterials for *in vivo* applications (Crapo et al., 2011).

### 3.2 Histological analysis of NWL and DWL

Histological analysis revealed that the DWL scaffolds maintained the native architecture of the tissue, with minimal disruption to the ECM components. This finding is consistent with previous studies demonstrating the efficacy of decellularization methods in preserving the structural integrity of plant-based biomaterials (Ahangar Salehani et al., 2023; Wang et al., 2020). It has been reported human cells showed good adhesion, proliferation, and function on the plant ECM scaffolds (Dikici et al., 2019; Gershlak et al., 2017; Harris, Lacombe, Liyanage, et al., 2021; Salehi et al., 2020). Therefore, by preserving the natural ECM structure, DWL may offers a supportive and instructive environment for cells, enhancing the overall effectiveness of skin tissue engineering strategies.

### 3.3 FTIR and Raman spectroscopy of NWL and DWL

**FIGURE 3:**
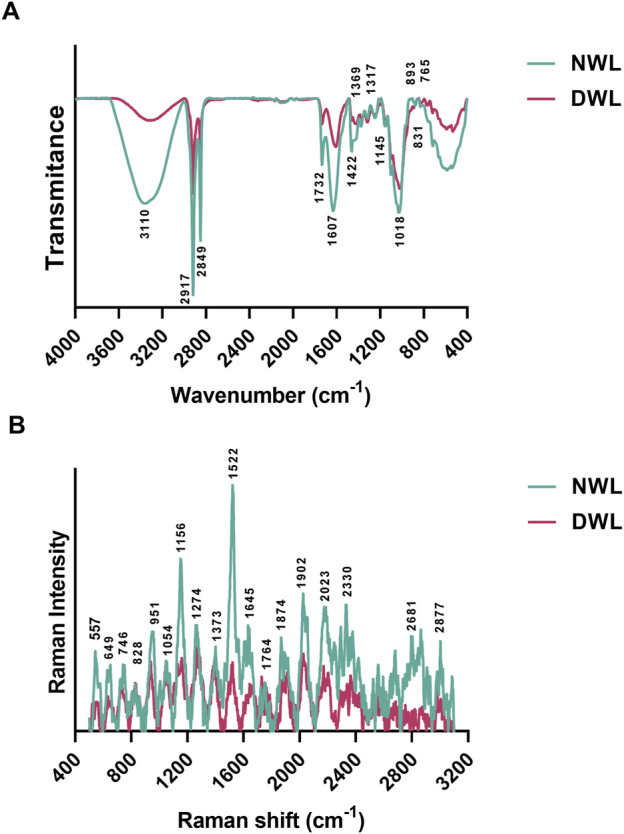
FTIR and Raman spectra of extracellular matrix contents for NWL and DWL samples [**(A, B)**, respectively].

FTIR and Raman spectra of NWL and DWL are illustrated in Figures 3A, B, respectively. Regarding FTIR peaks, the broad peak around 3,310 cm^-1^ is attributed to the stretching vibrations of O-H in the water or phenols structures (A. F. Harris, Lacombe, Liyanage, et al., 2021; Masek et al., 2019; Topală et al., 2020). Two sharp peaks at 2,917 and 2,849 cm^-1^ can be related to CH_2_ asymmetrical and symmetrical stretching of wax substances, respectively with some contribution from other macromolecules, such as carbohydrates (Harris, Lacombe, Liyanage, et al., 2021; Koochakzaei and Sabaghian, 2023). The peak at 1732 cm^−1^ is attributed to the C=O stretch vibration of esters. The peak at 1,607 cm^−1^ can be attributed to aromatic C=C stretch and/or asymmetric C-O stretch vibrations in COO- of lignin or asymmetric COO- of pectin (Harris, Lacombe, Liyanage, et al., 2021; Masek et al., 2019). The minor peak at 1,422 cm^−1^ is related to the bending vibration of CH_2_ in the structure of cellulose and also of hemicellulose, lignin, and pectin. Also, both mentioned peaks can correspond to the stretching vibrations of the aromatic rings within tannins (Koochakzaei and Sabaghian, 2023). The minor vibration at 1,369 cm^−1^ is associated with the C–H deformation of phenolic and aliphatic compounds. The peak at 1,317 can be related to the hydrolysable tannins (Koochakzaei and Sabaghian, 2023) or the CH_2_ bending vibration of cellulose and aliphatic molecules in the cuticle (Harris, Lacombe, Liyanage, et al., 2021). Minor peaks from 1,200 to 1,030 cm^−1^ can correspond to C-O-C glycosidic linkage vibrations, C-O stretching, and also, O-H deformation vibrations in secondary alcohols and phenols (Masek et al., 2019). The peak at 1,145 cm^−1^ can be related to the stretching vibration of C–O–C groups in non-cellulosic carbohydrates, such as hemicellulose and pectin (Harris, Lacombe, Liyanage, et al., 2021). The sharp peak at 1,018 cm^−1^ corresponds to the stretching vibration of C-O in the structure of carbohydrates, including cellulose, hemicellulose, and pectin (Harris, Lacombe, Liyanage, et al., 2021). The vibration at 893 can be related to β linkage of cellulose (Harris, Lacombe, Liyanage, et al., 2021). Two minor peaks at 831 and 765 can be attributed to the gallotannins, such as gallic acid (Koochakzaei and Sabaghian, 2023). Analysis of the DWL shows the existence of the mentioned component, however the intensity of peaks was decreased (Figure 3A).

Regarding Raman spectrum of NWL scaffolds, the peak at 557 cm⁻^1^ is attributed to the C–C stretching vibrations in cellulose (Schenzel and Fischer, 2001). The peak at 746 can be related to the carboxyl group in pectin (Gupta et al., 2020). The peak at 1,156 cm⁻^1^ is typically associated with the vibrational modes of carotenoids, similar to the peak at 1,522 cm⁻^1^ (Payne et al., 2024). Also, it can be due to asymmetric stretching vibration of C-C and C-O in cellulose (Schenzel and Fischer, 2001; Zhang, 2021). The peak at 1,274 cm⁻^1^ can be associated to aryl ether bonds of lignin (Gierlinger and Schwanninger, 2006; Zhang, 2021). The peak at 1,373 cm⁻^1^ can be attributed to HCC, HCO, and HOC bending of cellulose (Gierlinger and Schwanninger, 2006; Zhang, 2021). The peak at 1,645 cm⁻^1^ in a plant leaf is typically associated with the vibrational modes of protein amide I bonds (Payne and Kurouski, 2021). The peak at 1764 cm⁻^1^ in a plant leaf is typically associated with the vibrational modes of lipids, particularly those related to ester functional groups. The 2,877 cm⁻^1^ peak corresponds to the symmetric and asymmetric stretching vibrations of CH_2_ and CH_3_ groups within lipid molecules or cellulose and hemicellulose (Zhang, 2021). The intensity of peaks was lowered in the DWL scaffolds (Figure 3B).

As mentioned earlier, walnut leaves are rich in polyphenolic compounds, which have been reported to possess antioxidant, anti-inflammatory, and antimicrobial properties (Jahanban-Esfahlan et al., 2019; Sharma et al., 2022). The results from FTIR and Raman spectroscopy analyses revealed that some components remained after decellularization, suggesting that the decellularization process did not completely remove all the bioactive molecules and components present in the walnut leaf tissue. Similar findings have been reported in previous studies involving the decellularization of other plant leaves, such as spinach (Salehi et al., 2020), baby spinach (Harris, Lacombe, Liyanage, et al., 2021), and tomato (Ahmadian et al., 2023) leaves, that have demonstrated the retention of bioactive molecules after the decellularization process.

### 3.4 SEM and EDX analysis

**FIGURE 4:**
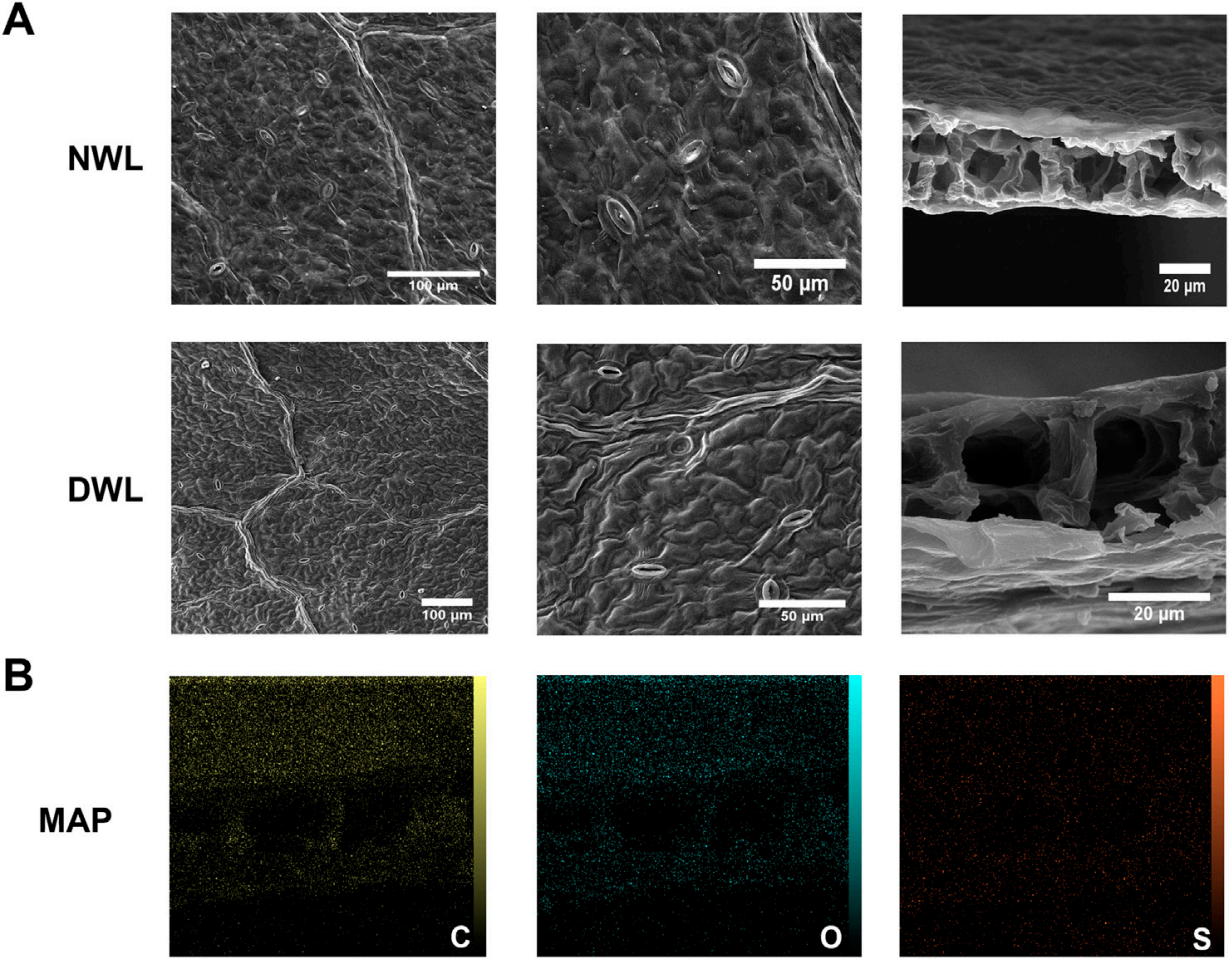
SEM photographs of NWL and DWL scaffolds **(A)**. EDX elemental maps of the DWL scaffold **(B** **)**.

SEM analysis revealed that walnut leaves have a proper vascular and porous structure. The observations indicated that the decellularization process did not adversely affect the normal structure of walnut leaves. Also, EDX elemental maps revealed a remnant of C, O, and S elements in the structure of DWL scaffolds (Figure 4). The extracellular matrix (ECM) is a complex network of proteins and polysaccharides that provides not only structural support but also critical biochemical and biomechanical cues for cell behavior. The retention of ECM in DWL scaffolds post-decellularization is a positive indicator of their potential to support cell attachment and proliferation. This is because the ECM components can interact with cell surface receptors, influencing cell adhesion, migration, differentiation, and ultimately, tissue formation (Wang et al., 2020).

In lined with our findings, previous studies have shown that decellularized scaffolds derived from various plant sources retain their ECM and support mammalian cell growth. For instance, decellularized spinach and banana leaves have been explored for their potential in tissue engineering due to their preserved ECM and vascular structures (Banavar et al., 2024; Harris, Lacombe, Liyanage, et al., 2021).

Therefore, the SEM and EDX analyses support the notion that DWL scaffolds, with their retained ECM and elemental composition, offer a promising platform for cell growth and tissue engineering applications. The ability of these scaffolds to mimic the natural cellular environment could lead to advancements in regenerative medicine, particularly in wound healing and tissue repair.

### 3.5 Surface roughness parameters

**FIGURE 5:**
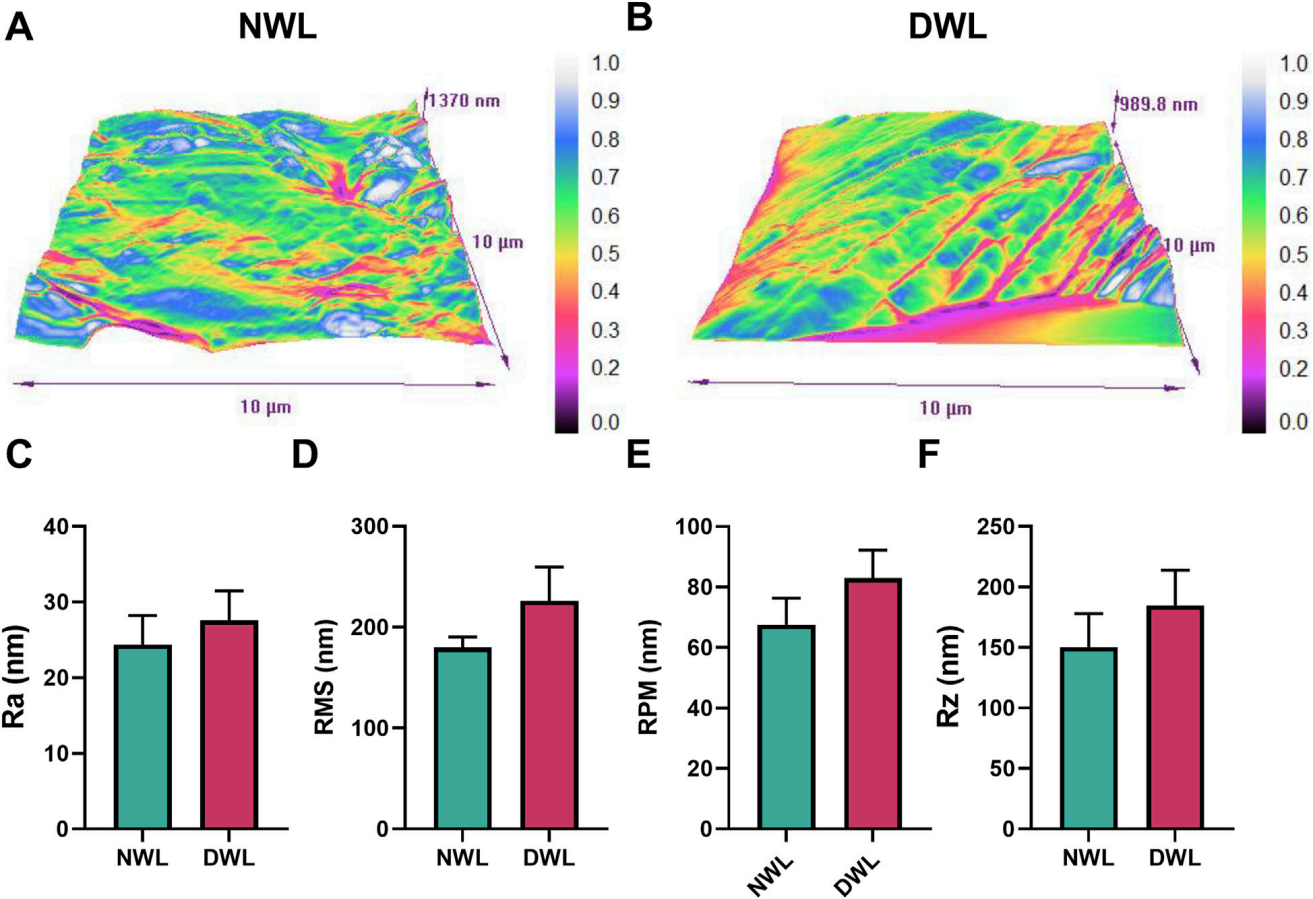
Surface roughness images **(A, B)** and properties [**(C–F)**, respectively] of NWL and DWL scaffolds.

To determine if the chemical decellularization process could affect the surface roughness parameters of the DWL, the roughness characteristics of both NWL and DWL samples were assessed using AFM. AFM-scanning images showed the heterogeneous surface topography of both NWL and DWL scaffolds (Figures 5A, B, respectively). Regarding roughness parameters including Ra, RMS, RPM, and Rz, they were 24.43 ± 3.82, 180 ± 10.30, 67.58 ± 8.75, and 150.3 ± 29.13, respectively for NWL samples and 27.63 ± 3.84, 226.2 ± 3.29, 82.96 ± 9.28, and 184.6 ± 29.13 for DWL scaffolds. The data did not have statistical differences between the NWL and DWL scaffolds (Figures 5C–F, respectively).

Adequate surface roughness of scaffolds is required for cell anchorage, proliferation, migration, differentiation, and protein absorption (Jafari et al., 2017; Salehi et al., 2020). Scaffold with nanometric scale surface roughness can facilitate cell interaction and adhesion (Dave and Gomes, 2019; Jafari et al., 2017). The average roughness (Ra) of DWL was 27.63 which is comparable with polymeric fibrous scaffolds (Jafari et al., 2017). Salehi et al. have reported a Ra of 11.5 nm for leaf spinach decellularized scaffold (Salehi et al., 2020). Also, our results showed that decellularization did not statistically affect the surface parameters, however, evidence on the effect of decellularization on the surface roughness of leaves is insufficient. Allan et al. have reported decellularization process has a smoothing effect on the surface roughness of decellularized grass scaffold (Allan et al., 2021). However, their inference is based on observational findings and did not report roughness parameters. On the other hand, the method of decellularization had some differences between the two studies.

### 3.6 Mechanical properties of NWL and DWL

**FIGURE 6:**
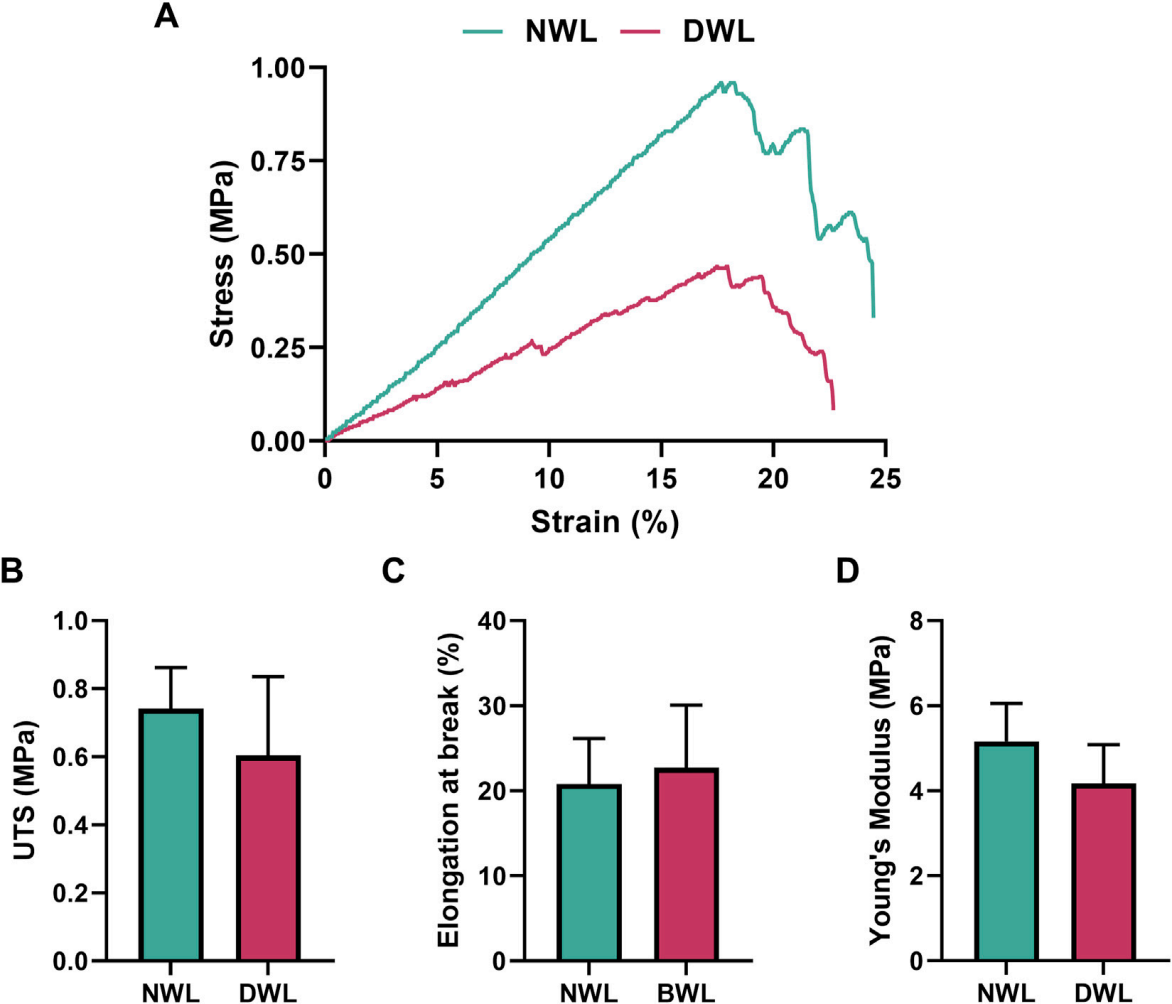
Stress-strain curve **(A)** and Mechanical properties of NWL and DWL scaffolds **(B–D)**.

Mechanical curves and properties of NWL and DWL scaffolds, including UTS, elongation at break, and Young’s modulus are represented in Figures 6 (A–D, respectively). For NWL, UTS was 0.74 ± 0.12 MPa, elongation at break was 20.82% ± 5.35%, and Young’s modulus was 5.16 ± 0.89 MPa, respectively. For DWL, these parameters were 0.60 ± 0.23 MPa, 22.73% ± 7.37%, and 4.17 ± 0.91 MPa, respectively. Statistical analyses indicated no significant differences between NWL and DWL scaffolds in terms of mechanical properties.

The lack of significant differences between NWL and DWL scaffolds in these mechanical properties suggests that the decellularization process preserves the essential mechanical characteristics of the walnut leaf scaffold. This is promising for biomedical applications, as it implies that the decellularized scaffolds maintain the necessary mechanical integrity for use as a supportive structure in tissue engineering (Harris, Lacombe and Zenhausern, 2021).

Despite the significant influence of mechanical properties on the cellular behavior of tissue-engineered scaffolds, only a few studies have characterized the mechanical strength of plant leaves before and after the decellularization process, and they remain understudied (Harris, Lacombe and Zenhausern, 2021). Ahangar Salehani et al. (2023) reported a value of 0.06 MPa for ultimate tensile strength (UTS) and an elasticity modulus of 4.99 MPa in decellularized olive leaves. Serkan Dikici (2024) found a UTS of 0.145 ± 0.007 MPa, an elongation at break of 21.9% ± 0.7%, and an elastic modulus of 0.843 ± 0.096 MPa in decellularized spinach leaves. Gershlak et al. (2017) reported a UTS of 0.05 MPa with a strain at failure of 0.06% in decellularized spinach leaves. These values align closely with our findings from mechanical evaluations.

### 3.7 Surface wettability, porosity, and swelling

**FIGURE 7:**
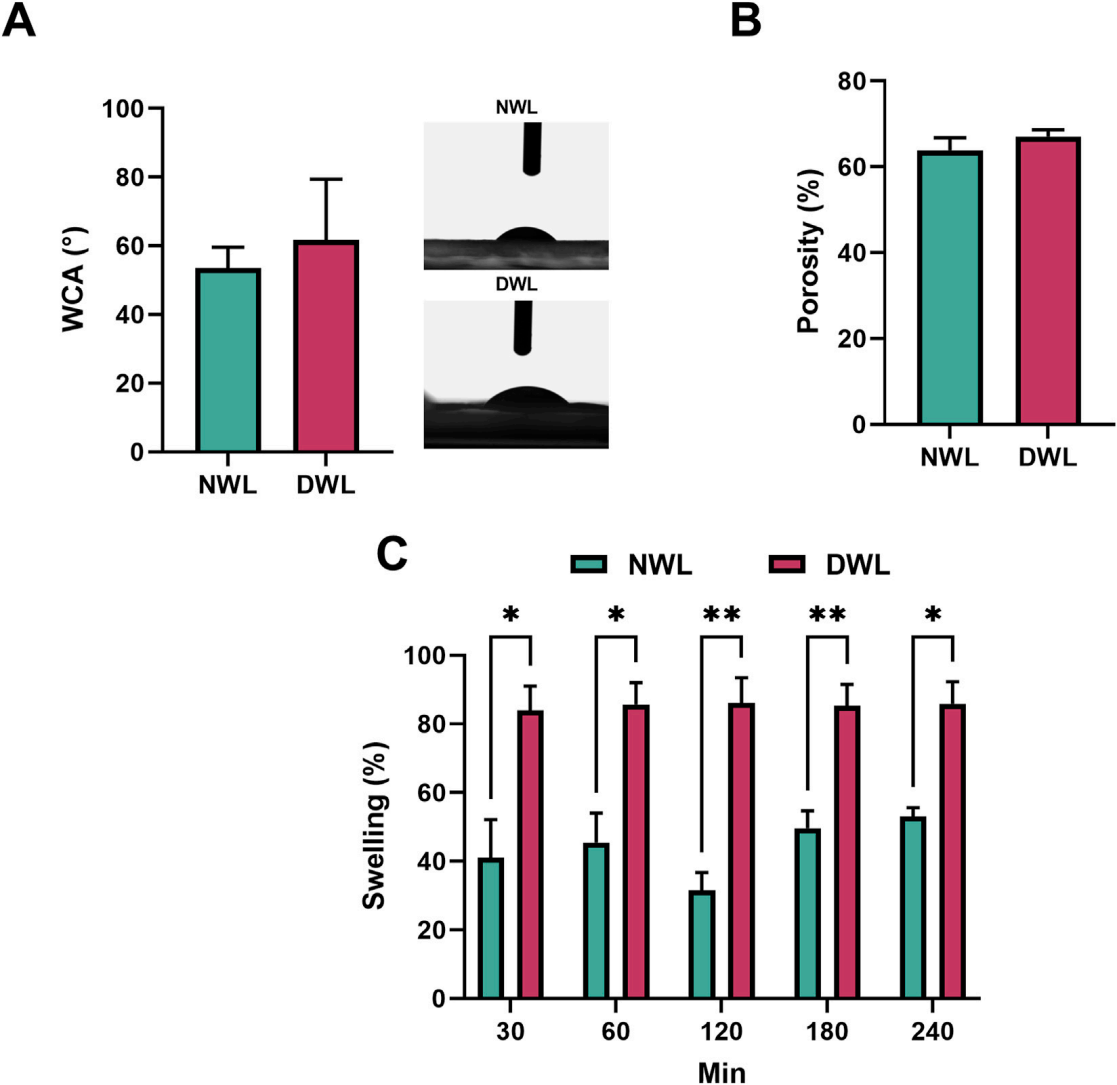
WCA and porosity of NWL and DWL scaffolds (**(A, B)**, respectively). Swelling behavior and its ratio in NWL and DWL scaffolds at different time points **(C)**.

Findings revealed that there was no significant difference in WCA and porosity percentage between NWL and DWL. They were 53.51 ± 6.12 and 63.85 ± 2.92 for NWL, and 61.73 ± 17.62 and 67.01 ± 1.65 for DWL scaffolds (Figures 7A, B, respectively). Figure 7C shows the swelling ratio of NWL and DWL scaffolds. It was observed that the swelling capacity of DWL was higher than the NWL scaffold in DW. Indeed, after 30 min, both scaffolds sharply reached a percentage of the swelling ratio of 41.01% ± 11.12% and 83.98% ± 7.06% for NWL and DWL, respectively. Thereafter, there was only a slight increase which reached equilibrium values with a mass swelling ratio of 53.03% ± 2.62% and 85.86% ± 6.43% at 240 min. The swelling ratio in DWL at all the time points was statistically (P < 0.05) higher than NWL scaffolds.

Interestingly, the NWL and DWL exhibited similar porosity, surface roughness parameters, and mechanical strength. These findings indicate that the decellularization process did not significantly alter the physical properties of the walnut leaves. This is important as it suggests that the decellularization process can be performed without compromising the structural integrity and mechanical properties of the walnut leaf scaffolds.

### 3.8 Cytotoxicity, cell viability, and cell morphology

**FIGURE 8:**
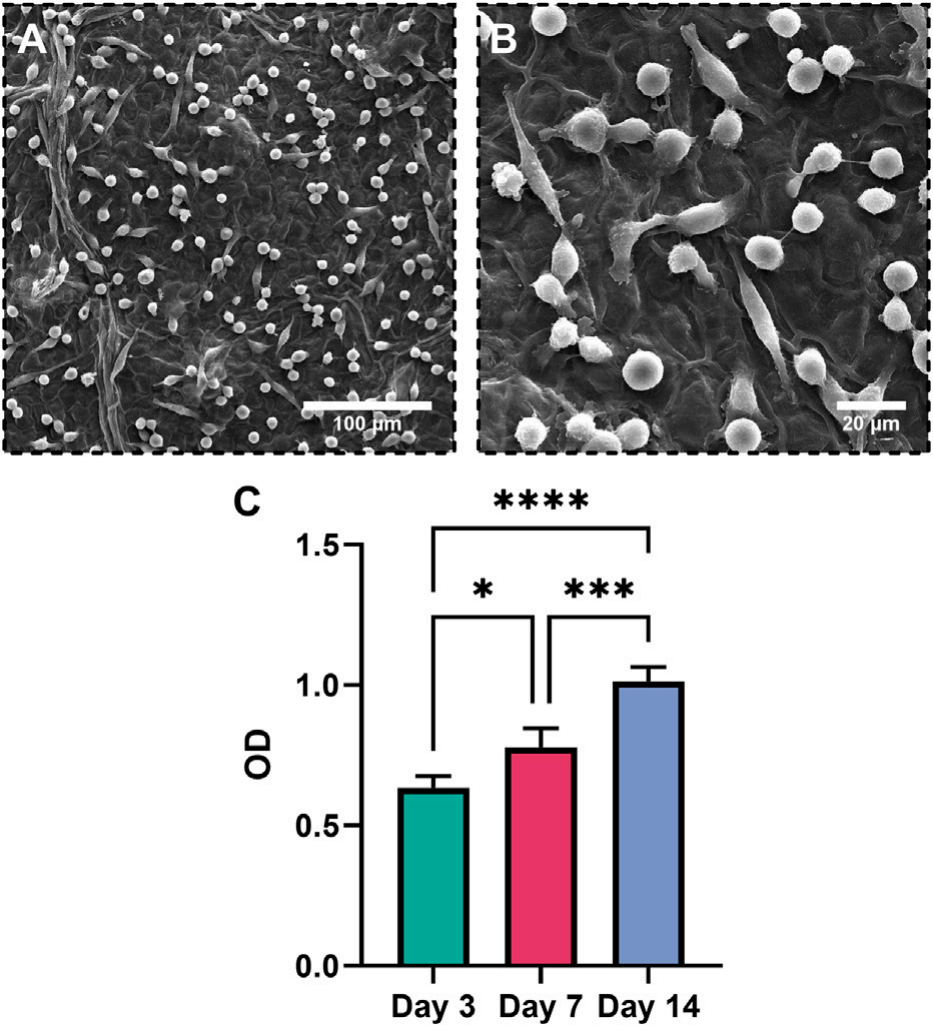
SEM photographs of hMSCs seeded on the DWL **(A, B)** and OD in different days **(C)**.

According to the MTT test (Figure 8C), the OD value of the hMSCs cultured on the DWL scaffold statistically increased on days 3 (P < 0.05), 7 (P < 0.05), and 14 (P < 0.0005) in comparison to the day 0. Increasing OD value correlates to the number, size, and metabolic activity of viable cells (Arifin et al., 2022; Khorraminejad-Shirazi et al., 2022). Hence, it can be concluded one or all of these parameters are influenced during culture of hMSCs on the DWL scaffold and also DWL is nontoxic and cytocompatible for hMSCs. The cytocompatibility of the DWL scaffold is also reflected in the SEM images (Figures 8A, B), where the hMSCs have adhered and well-distributed to the surface of the DWL scaffold and showed a mixture of circular and elongated cell morphologies. This suggests that the hMSCs are attaching well and interacting favorably with the scaffold. Such attachment is crucial for cell growth and proliferation (Liu et al., 2023; McCorry et al., 2016). It has been proved that cellulose based materials provide anchoring sites for cells (Seddiqi et al., 2021). However, further studies are needed to comprehensively assess its compatibility with living tissues. This pivotal characteristic is essential for ensuring the skin substitute can effectively support cell attachment, proliferation, and differentiation, ultimately leading to successful wound healing and tissue regeneration (Bruzauskaite et al., 2016; Solarte David et al., 2022).

### 3.9 *In vivo* biocompatibility and wound closure

**FIGURE 9:**
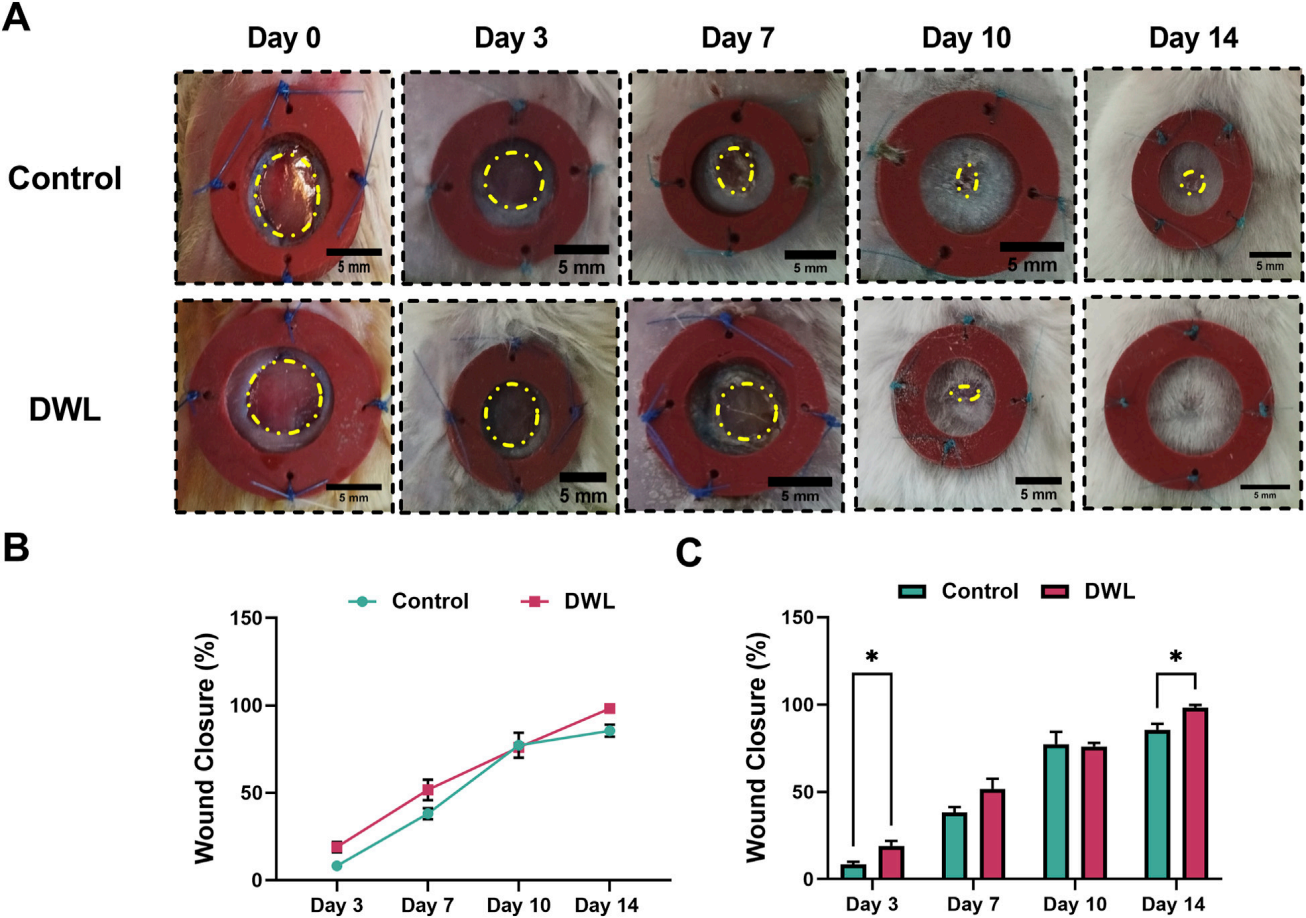
Photographs from experimental skin wounds **(A)** and percentage of wound closure in different days **(B, C)**.

**FIGURE 10:**
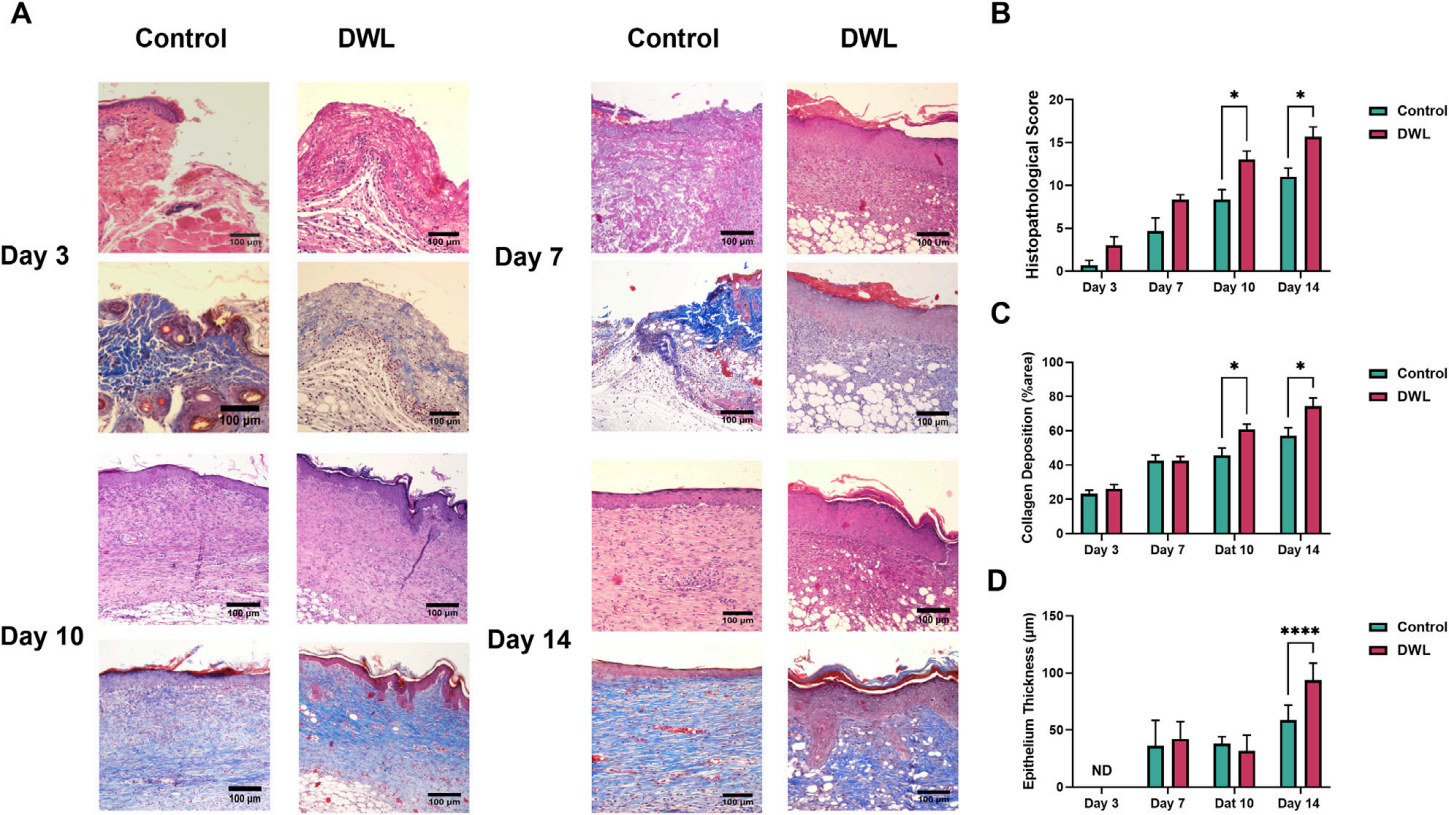
Histopathological photographs of wound samples on days 3, 7, 10, and 14 **(A)**. Histological score **(B)**, collagen deposition **(C)**, and epithelial thickness **(D)** in the histopathological samples of wounds on different days in the control and DWL groups.

Animal experimental investigations have indicated that DWL exhibits biocompatibility and can be applied as a wound dressing. Analysis of wound images (Figure 9A) revealed that on days 3 and 14 wound closure percent (Figures 9B, C) in animals dressed with DWL was statistically (P < 0.05) higher than the control animals. On the other hand, histopathological evaluations revealed that histopathological score and collagen deposition on days 10 and 14 and epithelium thickness on day 14 are statistically (P < 0.05 for histopathological score and collagen deposition and P < 0.0005 for epithelium thickness) improved compared to the control group (Figures 10A–D, respectively). *In vivo* evaluation using an excisional wound mice model demonstrated that the DWL scaffold significantly accelerated wound closure on days 3 and 14 compared to the control group. The wounds treated with DWL exhibited enhanced re-epithelialization, collagen deposition, and angiogenesis, indicating improved tissue regeneration and wound healing. Moreover, the histopathological score was significantly increased in wounds treated with DWL scaffolds, reflecting improved tissue organization, reduced inflammation, and enhanced wound healing compared to the control group.

These findings could be resulted from migration and proliferation of skin cells into the DWL scaffold or by release of bioactive molecules from DWL scaffold to the wound site. SEM analysis and MTT assay showed that hMSCs anchored and proliferated on the DWL scaffold. On the other hand, our study confirmed the presence of functional groups and bioactive molecules, including cellulose, hemi-cellulose, lignin, pectin, and more importantly carotenoids in DWL scaffolds. These components have various biological activities, such as immunomodulatory, anti-inflammatory, and antioxidant effects, antimicrobial properties, and accelerated wound healing potential (Hasanin, 2022; Liu et al., 2022; Naomi et al., 2020; Tarrahi et al., 2022; Ullah et al., 2022; Viana-Mendieta et al., 2022; Xiang et al., 2024).

There are only a few studies regarding the evaluation of biocompatibility and regenerative properties of decellularized plant-based scaffolds in animal models (Bai et al., 2021; Lee et al., 2019). Lee et al. (2019) found that a decellularized apple scaffold provided a good framework for bone-like tissue generation in a rat model of calvaria defect. Bai et al. (2021) reported that decellularized leaf and onion cellulose can be applied as vascular patches in a rat inferior vena cava patch venoplasty model.

While this experimental study provides valuable insights into the potential of DWL scaffolds for wound dressing applications, there are several limitations that require further investigation. Future studies should focus on optimizing the decellularization process, evaluating the long-term biocompatibility and degradation profile of the scaffolds, exploring the underlying mechanisms responsible for the observed therapeutic effects, and evaluating healing potential of DWL scaffold in more complex wound models, such as infected or diabetic ones.

## 4 Conclusion

Findings from the present study, demonstrate that DWL scaffolds retain some components as shown by FTIR and Raman spectroscopy, are biocompatible, and exhibit promising wound healing properties, including the ability to accelerate wound closure and increase the histopathological score in an excisional wound mice model. The retention of phytochemicals and the preservation of the ECM structure make DWL scaffolds attractive candidates for sustainable and cost-effective wound dressing applications. Future studies should focus on optimizing the decellularization process, conducting long-term *in vivo* studies and primary clinical trials, and exploring the underlying mechanisms responsible for the observed therapeutic effects.

## Data Availability

The original contributions presented in the study are included in the article/Supplementary Material, further inquiries can be directed to the corresponding authors.
